# Alternative Ready-To-Use Therapeutic Food Yields Less Recovery Than the Standard for Treating Acute Malnutrition in Children From Ghana

**DOI:** 10.9745/GHSP-D-19-00004

**Published:** 2019-06-24

**Authors:** Kristin Kohlmann, Meghan Callaghan-Gillespie, Julia M. Gauglitz, Matilda Steiner-Asiedu, Kwesi Saalia, Carly Edwards, Mark J. Manary

**Affiliations:** a Department of Pediatrics, Washington University, St. Louis, MO, USA.; b Collaborative Mass Spectrometry Innovation Center, Skaggs School of Pharmacy and Pharmaceutical Sciences, University of California, San Diego, San Diego, CA, USA.; c Department of Nutrition and Food Science, University of Ghana, Legon, Ghana.; d Project Peanut Butter, Kumasi, Ghana.; e Children's Nutrition Research Center, Baylor College of Medicine, Houston, TX, USA.

## Abstract

**Background:**

Only 20% of children with severe acute malnutrition (SAM) have access to ready-to-use therapeutic food (RUTF), and RUTF cost limits its accessibility.

**Methods:**

This randomized, double-blind controlled study involved a clinical equivalence trial comparing the effectiveness of an alternative RUTF with standard RUTF in the home-based treatment of uncomplicated SAM and moderate malnutrition in Ghanaian children aged 6 to 59 months. The primary outcome was recovery, equivalence was defined as being within 5 percentage points of the control group, and an intention-to-treat analysis was used. Alternative RUTF was composed of whey protein, soybeans, peanuts, sorghum, milk, sugar, and vegetable oil. Standard RUTF included peanuts, milk, sugar, and vegetable oil. The cost of alternative RUTF ingredients was 14% less than standard RUTF. Untargeted metabolomics was used to characterize the bioactive metabolites in the RUTFs.

**Results:**

Of the 1,270 children treated for SAM or moderate malnutrition, 554 of 628 (88%) receiving alternative RUTF recovered (95% confidence interval [CI]=85% to 90%) and 516 of 642 (80%) receiving standard RUTF recovered (95% CI=77% to 83%). The difference in recovery was 7.7% (95% CI=3.7% to 11.7%). Among the 401 children with SAM, the recovery rate was 130 of 199 (65%) with alternative RUTF and 156 of 202 (77%) with standard RUTF (*P*=.01). The default rate in SAM was 60 of 199 (30%) for alternative RUTF and 41 of 202 (20%) for standard RUTF (*P*=.04). Children enrolled with SAM who received alternative RUTF had less daily weight gain than those fed standard RUTF (2.4 ± 2.4 g/kg vs. 2.9 ± 2.6 g/kg, respectively; *P*<.05). Among children with moderate wasting, recovery rates were lower for alternative RUTF, 386 of 443 (87%), than standard RUTF, 397 of 426 (93%) (*P*=.003). More isoflavone metabolites were found in alternative RUTF than in the standard.

**Conclusion:**

The lower-cost alternative RUTF was less effective than standard RUTF in the treatment of severe and moderate malnutrition in Ghana.

## Abstract (precis)

In Ghana, an alternative ready-to-use food (RUTF) formulation that met all specifications was not as good as standard RUTF in affecting recovery from acute malnutrition among children aged 6 to 59 months.

## INTRODUCTION

In sub-Saharan Africa, 17 million children under 5 are wasted, which is defined as having a weight-for-length *z* score (WLZ) < 2 standard deviations (SD) below the mean World Health Organization (WHO) Child Growth Standards.^1^ Wasting leaves these children with an increased risk of illness and death.^2^ A large fraction of wasting occurs in children aged 6 to 24 months, a dynamic period of physical and neurological development.^3^ The majority of wasted children do not live in communities beset with emergencies, but rather come from the poorest segments of all countries. In general, these countries do not have the resources from donated or endogenous sources to sponsor widespread feeding and education programs to combat wasting. Effective and cost-efficient solutions to reduce wasting outside of acute emergencies will be necessary to achieve the Sustainable Development Goals.^2^

Among children under 5 years of age in Ghana, the prevalence of wasting is about 5%.^2^ Treatment for severe wasting, which is defined as WLZ ≤ −3 SD below the mean, is available in the northernmost regions of Ghana, where the density of SAM is greatest but where only 17% of the population reside. Treatment of moderate wasting, defined by WLZ > −3 and ≤ −2 and known as moderate acute malnutrition (MAM), is almost entirely unavailable in Ghana.

Home-based therapy with ready-to-use therapeutic food (RUTF) for children with SAM has revolutionized the management of wasted children, offering a superior alternative to inpatient treatment.^4^^,^^5^ Unfortunately, RUTF reaches only about 15% of the children worldwide who need it. Despite being highly cost-effective, SAM treatment is expensive in absolute terms, with a cost of US$150 to $200 per child, and in Ghana, one limited study estimated the cost of treating SAM to be $805 per child.^6^^,^^7^ Worldwide, standard RUTF (S-RUTF) is an expensive component of treatment, costing $47 to $61 per child treated.^8^ S-RUTF is composed of 25% skimmed milk powder and 27% peanut paste, a vegetable oil rich in omega-3 polyunsaturated fatty acids such as canola and sugar.

In 2013, our team initiated a multinational alternative RUTF (A-RUTF) formulation project with the aim to reduce the cost of RUTF, and in doing so, enable the existing resource envelope for SAM to be used to treat more children. The work began with a comprehensive literature and nutrient database analysis and subsequent development of a food formulation linear programming (LP) tool.^9^ The LP tool is a conventional computer database program that lists all potential ingredients, nutritional compositions, prices, and country-specific availability. The tool has default nutrient constraints that align the formulations with the international RUTF nutrient specifications and food safety guidelines.^10^ The tool also allows for ingredient constraints, which supports organoleptic optimization.^9^^,^^11^ It has been successfully used by our investigative team to create country-specific locally produced A-RUTF formulations for Ghana, Ethiopia, Pakistan, and India that were proven to be feasible, acceptable, and without adverse side effects in formal acceptability trials.^12^ However, the relative effectiveness of an A-RUTF to S-RUTF has yet to be shown.

This article describes the operation and results from a randomized, double-blind controlled clinical trial testing the hypothesis that a locally produced A-RUTF was equivalent to S-RUTF for the treatment of uncomplicated SAM and MAM, in the Brong Ahafo region of Ghana.

We tested whether a locally produced A-RUTF was equivalent to S-RUTF for treating acute malnutrition in Ghana.

## METHODS

### Subjects and Setting

Eligible children were between 6 and 59 months of age and experiencing acute malnutrition. SAM was defined as WLZ ≤ −3, or having a mid-upper arm circumference (MUAC) of <11.5 cm or bipedal edema. MAM was defined as not having SAM and having WLZ ≤ −2 or MUAC of <12.5 cm. In addition to meeting the anthropometric criteria, children were required to consume 30 g of RUTF in a supervised setting to be eligible for enrollment. Children were excluded if they were involved in another research trial or feeding program, had a chronic debilitating illness (e.g., cerebral palsy), or had a history of peanut or milk allergy.

Informed consent was obtained from the primary caregiver of the participant and documented by the caregiver's signature or thumbprint. The study received ethical approval from the Washington University in St. Louis Institutional Review Board, the Noguchi Memorial Institute for Medical Research Institutional Review Board, and the Ghana Health Service.

Study participants were recruited at 29 clinics throughout 5 districts in the Brong Ahafo region of Ghana. The Brong Ahafo region is the second largest region in Ghana and has the sixth largest population at 2.3 million.^13^ In 2011, the under-5 mortality rate in the region was 108 deaths per 1,000 live births, 32% higher than the national under-5 mortality rate.^14^ Although wasting rates in the latest Demographic and Health Survey showed a national decline, regional trends indicated that rates in the Brong Ahafo region had increased.^15^ In addition, 16% of all households in this region are considered food insecure.^16^

Study participants were recruited in the Brong Ahafo region, where the under-5 mortality was 32% higher than the national rate in 2011.

### Study Design

This randomized, double-blind controlled study was based on a clinical equivalence trial of treating acute malnutrition with 1 of 2 therapeutic foods, A-RUTF or S-RUTF. The primary outcome was recovery, defined as having achieved either WLZ > −2 or MUAC >12.4 cm at any point during the treatment. Equivalence was chosen as being within 5 percentage points of the control group. Secondary outcomes were rates of weight and MUAC gain, the number of visits before recovery, cost of RUTF per child recovered, and adverse events. The sample size was estimated to be 1,262 children, which gave the comparison sufficient power to detect a 5% difference in recovery, assuming the control group achieved recovery rate of 85% using an equivalence design. The assumption that recovery would be 85% overall for the treatment of MAM and SAM was based on our trials in Malawi.^5^ The trial was publicly registered as ISRCTN14788669.

### Participation and Data Collection

All participants were randomized to receive either A-RUTF or S-RUTF via a closed envelope technique. Allocation of the food intervention was conducted by a nurse who had the participant's caregiver draw an opaque envelope containing 1 of 4 colors. Each color corresponded to a type of RUTF. Both the research team and study participants were blinded to color assignments.

All participants were randomized to receive A-RUTF or S-RUTF; neither they nor the research team knew which was received.

Management of MAM and SAM followed an optimized protocol that incorporated many elements from the community management of acute malnutrition (CMAM), which is described in Table 1. Notable deviations from CMAM were (1) visits were fortnightly instead of weekly, (2) the ration of RUTF for SAM was reduced as the child gained weight, (3) MAM children were given supplementary food in addition to counseling, and (4) exit criteria for the study were achievement of MUAC >12.4 cm on a single occasion or completion of 12 weeks of feeding, instead of requiring 3 occasions with MUAC >12.4 cm.

**TABLE 1.** Comparison of Project Peanut Butter and Ghana Health Service Malnutrition Management Protocols in Brong Ahafo, Ghana

	Project Peanut Butter Protocol	Ghana Health Service CMAM Protocol
**SAM enrollment criteria**	MUAC <11.5 cm or WLZ < −3 SD Bilateral pitting edema	MUAC <11.5 cm or WLZ below −3 SD Bilateral pitting edema
MAM treatment MUAC ≥11.5 cm, <12.5 cm WLZ between −2 and −3 SD	Enrolled and treated with RUTF	Increased nutrition counseling during CWC
**RUTF dosage**	150 kcal/kg/day for SAM 75 kcal/kg/day for MAM	200 kcal/kg/day for SAM
**Follow-up**	Biweekly	Weekly
**Graduation criteria**	MUAC >12.4 cm, or WFL > −2 SD for 1 visit (2 weeks)	MUAC >12.4 cm, 3 consecutive weeks No edema, 3 consecutive weeks
**Discharge criteria**	3 consecutive visits missed (6 weeks)	3 consecutive weeks missed
**Maximum duration of treatment**	12 weeks	16 weeks

Abbreviations: CMAM, community management of acute malnutrition; CWC, Child Welfare Clinics; MAM, moderate acute malnutrition; MUAC, mid-upper arm circumference; RUTF, ready-to-use food; SAM, severe acute malnutrition; WLZ, weight-for-length *z* score.

The children had MUAC, weight, and length measured upon enrollment. MUAC was measured on the left arm with a standard insertion tape to the nearest 0.1 cm (TALC, Herts, UK); weight was measured to the nearest 5 g using an electronic scale (Seca 334, Hamburg Germany, calibrated weekly); and recumbent length was measured in triplicate to the nearest 0.2 cm, using a rigid length board (Seca 417 length board, Hamburg, Germany). The staff received standardized training every 8 weeks in the measurement of edema and anthropometry by a senior clinician, and 10% of the field measurements were rechecked in the field for quality purposes. During the initial visit, demographic and health information were recorded, and a 2-week supply of their assigned RUTF was dispensed. The dosage of RUTF provided a daily intake of about 150 kcal/kg for SAM participants and about 75 kcal/kg for MAM participants. The daily SAM ration provided about 100% of the child's needs for growth and maintenance and was typically about 200 g. The daily MAM ration provided about 60% of the child's needs for growth and maintenance and was typically about 100 g. Caregivers and study participants were asked to return every 2 weeks for follow-up. At follow-up, caregivers reported on the child's clinical symptoms, anthropometric measurements were taken, and additional RUTF was distributed for those that remained wasted. The dosage of RUTF distributed at each follow-up visit was determined by the child's current weight. As SAM participants began to recover and reached a MUAC ≥11.5 cm, they were transitioned to the MAM dosage of 75 kcal/kg/day of their assigned RUTF. No additional food rations were given when subjects reached an outcome, nor were the children asked to return for follow-up at regular intervals.

The study was implemented by trained nurses working for Project Peanut Butter, a registered NGO in Ghana. A research associate from Washington University resided in Ghana for the purposes of implementing the study as well. Health center facilities were used as locations where malnutrition treatment services were given, but Project Peanut Butter ensured that RUTF was always available and research staff were always present on the appointed days to deliver service. This was done so that the results of the trial could be interpreted as a comparison of the effectiveness of 2 types of RUTF, without bias due to barriers to consistent implementation.

### Study Foods

Both RUTFs were produced at Project Peanut Butter in Kumasi, Ghana, a certified local supplier. Both RUTF formulations met the nutritional specifications and microbiological requirements for RUTF set forth by United Nations agencies in 2007 and underwent safety testing for aflatoxin and microbial contamination at Eurofins Scientific Inc. (Des Moines, Indiana, USA).^10^ The S-RUTF contained peanut paste, sugar, nonfat dried milk, vegetable oil, a premix containing concentrated minerals and vitamins, and an emulsifier. The A-RUTF replaced about half the amount of peanut with locally available soybean and sorghum flour, and the 50% of protein from dairy per United Nations specification came from a combination of whey protein concentrate 34 and nonfat dried milk. A-RUTF also included canola oil, sugar, a vitamin and mineral premix, as well as less nonnutritive emulsifier (Table 2).

**TABLE 2.** Ingredient and Nutrient Composition of Study Foods^a^

Ingredient/Nutrient	Alternative-RUTF	Standard-RUTF
**Ingredient**		
Cereal/grain, sorghum, g/100 g	9.00	—
Legume, g/100 g		
Groundnut	14.00	27.00
Soybean	2.00	—
Milk, g/100 g		
Dry, nonfat, regular, without added vitamin A and vitamin D	5.00	25.00
Whey protein concentrate 34%	20.18	—
Oil, g/100 g		
Canola	20.50	—
Palm	—	15.48
Soybean	—	2.92
Sugar, g/100 g	25.00	24.64
Micronutrient and vitamin premix, g/100 g	2.92	2.96
Emulsifier, g/100 g	1.40	2.00
**Nutrient**		
Energy, kcal/100 g	560	559
Protein, g/100 g	14.5	15.8
Lipids, g/100 g	29.2	33.0
n-6 fatty acids, g/100 g	6.3	5.7
n-3 fatty acids, g/100 g	1.9	0.03

Abbreviation: RUTF, ready-to-use therapeutic food.

a Both foods were a soft, brown, homogeneous paste with small granules perceptible to the tongue. They were packaged in identical, unlabeled metalized polyethylene terephthalate sachets with the only marking being a colored dot to indicate the type of RUTF.

Both RUTF formulations used in the study met nutritional and microbiological requirements and underwent safety testing.

Whenever study food was given to children, the nurses counseled the caregivers to feed the RUTF in whatever manner the child would readily accept it, which was most often sucking the food out of the flexible package from a small tear. Caregivers were also counseled to feed the RUTF strictly to the malnourished child and not to share or sell the RUTF.

The ingredient cost of A-RUTF was US$1.90/kg compared with $2.20/kg for the S-RUTF, a 14% cost reduction in ingredients. This reduction was largely achieved by substituting the less expensive sorghum and soy for peanut. Ingredient prices were estimated using the LP tool, which employed a modeling method that determined the median commodity prices in 2012 in Ghana from a comprehensive variety of sources, including accounting for transportation and taxes. The price variation seen in the subsequent 5 years was then added to the model to estimate “typical” prices for the ingredients.

Protein quality was calculated to better characterize A-RUTF and S-RUTF. The Digestible Indispensable Amino Acid Score (DIAAS) method with the reference population being healthy children aged 1–3 years was used to calculate protein quality.^17^ In addition, the DIAAS was recalculated using malnourished children in a phase of rapid catch-up growth as a reference population.^18^

### Coverage Survey

To determine coverage of MAM and SAM children receiving RUTF feeding (i.e., the proportion of children with acute malnutrition who were accessing services), we used the simplified lot quality assurance sampling evaluation of access and coverage (SLEAC) method.^19^ The coverage survey was conducted as a routine measure of program effectiveness, which allowed us to understand if the research feeding achieved similar coverage as operational programs in sub-Saharan Africa.

### Metabolomics Analysis

To characterize the nonnutritive components of the RUTFs, which might contribute to the clinical effect, untargeted metabolomics analyses were conducted. A-RUTF and S-RUTF were extracted to a final concentration of 1 μg/μL in 50% methanol and 95% ethanol for untargeted metabolite analysis. Data were acquired for each sample in triplicate using an ultra-high performance liquid chromatography–tandem mass spectrometry system (UltiMate 3000 UHPLC system [Thermo Scientific, Waltham, MA, USA] coupled to a Maxis Q-TOF mass spectrometer [Bruker Daltonics, Bremen, Germany]), using electrospray ionization in positive mode and a reverse phase C18 column (Kinetex, 100 × 2.1 mm, 1.7-μm particle size, 100-Å pore size; Phenomenex, Torrance, CA, USA). Raw data files were converted to mzXML format using Bruker DataAnalysis software after lock mass correction (*m/z*=622.0290; Hexakis [SynQuest Laboratories, Alachua, FL, USA]) and analyzed with molecular networking and library spectral matching using the web-based platform GNPS (https://gnps.ucsd.edu). The analysis is available at https://gnps.ucsd.edu/ProteoSAFe/status.jsp?task=a474e2ed686f43d7b2946a53225495c2.

### Data Analysis

Data were double entered into a Microsoft Access database and discrepant values corrected by reviewing the original data collection cards. For children older than 24 months, height was estimated from the measured length by subtracting 1.5 cm from the length.^20^ *Z* scores were calculated using the WHO Anthroplus version 1.0.4 (WHO, Geneva), based on the 2006 WHO Child Growth Standards.^21^ Rates of weight gain were calculated for the first 4 weeks of treatment by dividing weight gain in grams by the enrollment weight in kilograms and the days of treatment between measurements. Mean daily MUAC gain was also calculated for the first 4 weeks of treatment by dividing MUAC gain in millimeters by days of treatment between measurements.

Data were analyzed by using SPSS Statistics software (version 25.0; IBM Corp., Armonk, NY, USA). Summary statistics were calculated for the participants as mean ± SD for continuous parameters and n (%) for categorical parameters. Analyses were done by intention to treat (ITT) for which defaulters were considered to be failures in accordance with the Sphere Standards.^22^ In accordance with the trial designation as an equivalence trial, the 95% confidence intervals (CIs) around the recovery rates were calculated to determine if there was overlap between the groups and the difference was compared to determine if it exceeded the threshold of 5 percentage points.

Subgroup analyses were performed on children with SAM and MAM. For secondary and subgroup outcomes treatment groups were compared using the Student's test or Mann-Whitney *U* test for continuous variables and Fisher's exact test for categorical measures.

## RESULTS

**FIGURE 1:**
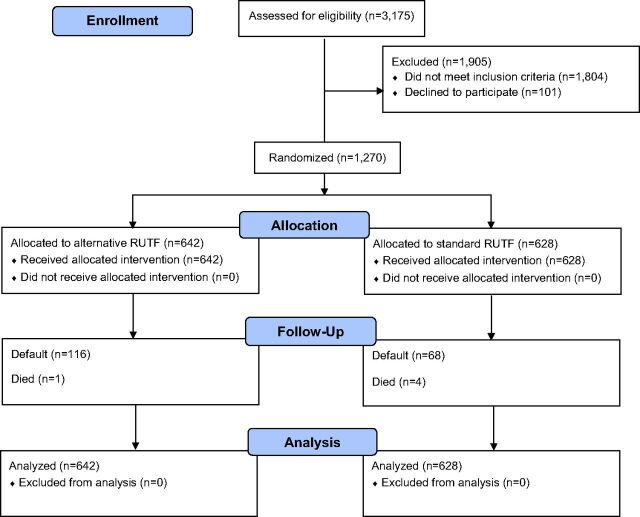
CONSORT Flow Diagram Abbreviation: RUTF, ready-to-use therapeutic food.

A total of 1,270 children were enrolled in the study from February 2017 to February 2018 (Figure 1). Of these, 401 were diagnosed with SAM and were assigned to receive either A-RUTF (n=199) or S-RUTF (n=202); 869 children were diagnosed with MAM and were assigned to receive either A-RUTF (n=443) or S-RUTF (n=426). The baseline characteristics for each study group were similar (Table 3). For the children with SAM, 157 were designated by both MUAC and weight-for-length, 178 only by MUAC, 63 only by weight-for-length, and 3 only by having edema. For the children with MAM, 320 were designated by both MUAC and weight-for-length, 378 only by MUAC, and 171 only by weight-for-length.

**TABLE 3.** Characteristics of Study Children at Enrollment

Characteristic	Severe Acute Malnutrition		Moderate Acute Malnutrition	
	A-RUTF (n=199)	S-RUTF (n=202)	A-RUTF (n=443)	S-RUTF (n=426)
Male, No. (%)	88 (44.2)	94 (46.5)	189 (42.7)	163 (38.3)
Age, months, mean (SD)	14.38 (8.0)	13.25 (7.6)	15.79 (9.3)	13.89 (7.2)
Roof made of metal, No. (%)	152 (76.4)	153 (75.7)	358 (80.8)	346 (81.2)
Animals sleep with child, No. (%)	120 (60.3)	127 (62.9)	259 (58.5)	275 (64.6)
Electricity in home, No. (%)	111 (55.8)	113 (55.9)	285 (65.2)	267 (62.7)
Clean water source, No. (%)	83 (41.7)	91 (45.0)	206 (46.5)	206 (48.4)
Edema, No. (%)	5 (2.5)	3 (1.5)	—	—
Mid-upper arm circumference, cm, mean (SD)	11.1 (0.9)	11.0 (0.8)	12.2 (0.4)	12.2 (0.4)
Weight, kg, mean (SD)	6.28 (1.3)	6.12 (1.2)	7.34 (1.3)	7.19 (1.2)
Length, cm, mean (SD)	69.8 (7.5)	68.5 (6.6)	72.4 (7.2)	71.2 (6.6)
Weight-for-length, *z* score, mean (SD)	-3.16 (0.8)	-3.07 (0.9)	−2.08 (0.6)	−1.97 (0.6)
Length-for-age, *z* score, mean (SD)	-2.56 (1.4)	-2.64 (1.4)	−1.98 (1.2)	−1.80 (1.1)
Weight-for-age, *z* score, mean (SD)	-3.65 (0.9)	-3.64 (1.0)	−2.59 (0.7)	−2.41 (0.9)

Abbreviations: A-RUTF, alternative ready-to-use therapeutic food; SD, standard deviation; S-RUTF, standard ready-to-use therapeutic food.

A total of 1,270 children were enrolled in the study from February 2017 to February 2018.

Among SAM and MAM children receiving A-RUTF, 516 of 642 recovered (80.4%, 95% CI=77.1% to 83.3%) (Table 4). Among children receiving S-RUTF, 553 of 628 recovered (88.1%, 95% CI=85.3% to 90.4%). The difference in recovery rates was 7.7 percentage points (95% CI=3.7 to 11.7 percentage points).

**TABLE 4.** Comparison of Outcomes Between Assigned Treatment Food for Ghanaian Children With Severe Acute Malnutrition and Moderate Acute Malnutrition

Outcome	Assigned A-RUTF	Assigned S-RUTF	*P* Value^a^
**All study participants**	**n=642**	**n=628**	
Defaulted,^b^ No. (%)	116 (18.1)	68 (10.8)	<.001
Died, No. (%)	1 (0.2)	4 (0.6)	.21
Recovered, No. (%)	516 (80.4)	554 (88.2)	<.001
Remained malnourished, No. (%)	9 (1.4)	2 (0.3)	.06
Rate of weight gain,^c^ g/kg/d, mean (SD)	1.88 (1.8)	2.04 (2.0)	.31
Rate of MUAC gain,^c^ mm/d, mean (SD)	0.16 (0.2)	0.18 (0.2)	.04
**Participants with SAM**	**n=199**	**n=202**	
Defaulted,^b^ No. (%)	60 (30.1)	41 (20.3)	.03
Died, No. (%)	1 (0.5)	3 (1.5)	.62
Recovered, No. (%)	130 (65.3)	156 (77.2)	.01
Remained malnourished, No. (%)	8 (4.0)	2 (1.0)	.06
Rate of weight gain,^c^ g/kg/d, mean (SD)	2.40 (2.4)	2.90 (2.6)	.04
Rate of MUAC gain,^c^ mm/d, mean (SD)	0.20 (0.2)	0.25 (0.2)	.047
**Participants with MAM**	**n=443**	**n=426**	
Defaulted,^b^ No. (%)	56 (12.6)	27 (6.3)	.002
Died, No. (%)	0 (0.0)	1 (0.2)	>.99
Recovered, No. (%)	386 (87.1)	398 (93.4)	.003
Remained malnourished, No. (%)	1 (0.2)	0 (0)	>.99
Rate of weight gain,^c^ g/kg/d, mean (SD)	1.66 (1.5)	1.61 (1.5)	.62
Rate of MUAC gain,^c^ mm/d, mean (SD)	0.13 (0.2)	0.14 (0.2)	.29

Abbreviations: A-RUTF, alternative ready-to-use therapeutic food; MAM, moderate acute malnutrition; MUAC, mid-upper arm circumference; SAM, severe acute malnutrition; S-RUTF, standard ready-to-use therapeutic food.

a Statistical comparisons made using Student's *t* test for continuous parameters and Fisher's exact test for categorical parameters.

b Defaulters were treated as unrecovered in the calculation of recovery rates.

c Calculated for the first 4 weeks of treatment.

The protein content in 100 g of S-RUTF was 1.25 g higher than in 100 g of A-RUTF, although both foods were within current international agency specifications and 50% of protein was from a dairy source (Table 2).^10^ S-RUTF also had a lipid content that was 2.8 g more than A-RUTF per 100 g of RUTF. Protein quality as determined by DIAAS using healthy children as a reference population was 107 for A-RUTF and 109 for S-RUTF. Protein quality using malnourished children as a reference population was 85 for A-RUTF and 84 for S-RUTF.

**FIGURE 2:**
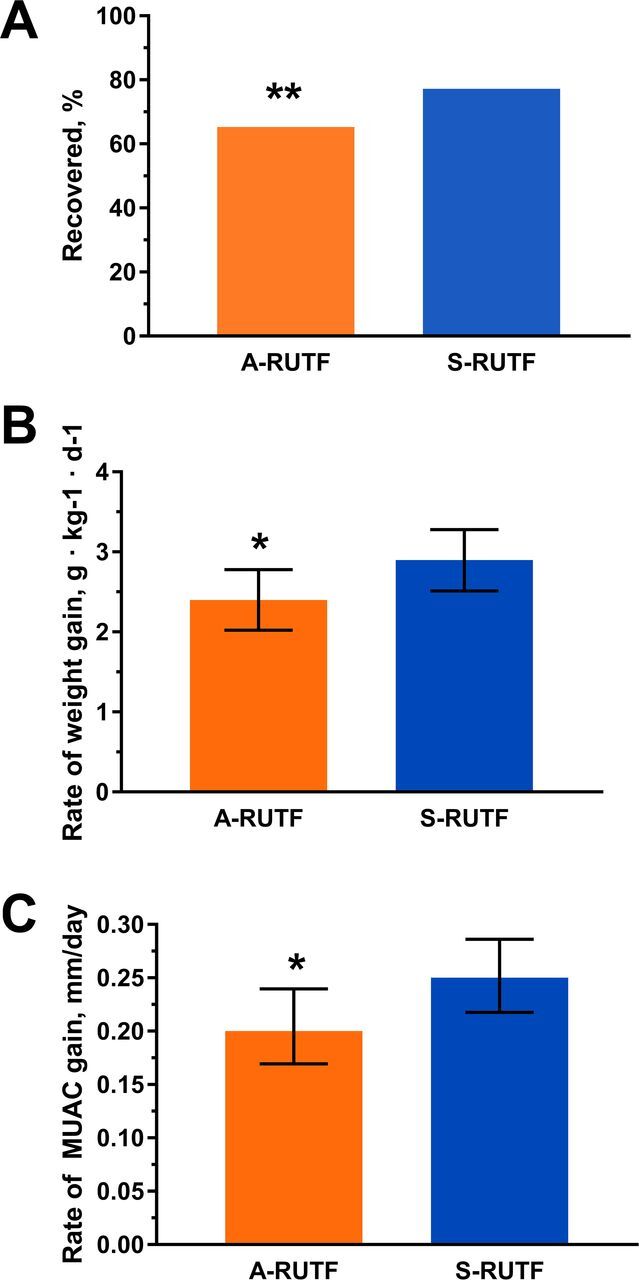
Comparison of Outcomes Between A-RUTF and S-RUTF Among Ghanaian Children With Severe Acute Malnutrition Abbreviations: A-RUTF, alternative ready-to-use therapeutic food; CI, confidence interval; S-RUTF, standard ready-to-use therapeutic food. A: Recovery rates for A-RUTF and S-RUTF compared with intention-to-treat analysis (Fisher's exact test, **P≤.01). B: Median rate of weight gain during the first 4 weeks of treatment for A-RUTF and S-RUTF; error bar indicates 95% CIs, *P≤.05. C: Median rate of MUAC gain during first 4 weeks of treatment for A-RUTF and S-RUTF; error bar indicates 95% CIs, Mann-Whitney *U* test, *P≤.05.

Among SAM children, recovery was seen in 130 of 199 (65.3%) for those receiving A-RUTF and was 156 of 202 (77.2%; *P*=.01) for those receiving S-RUTF (Table 4, Figure 2); defaults were not considered to be recovered in our ITT analysis. Children with SAM receiving A-RUTF had lower rates of gain for both weight and MUAC (Figure 2).

A-RUTF yielded lower rates of gain for weight and MUAC and a lower likelihood of recovery among children with SAM.

**FIGURE 3:**
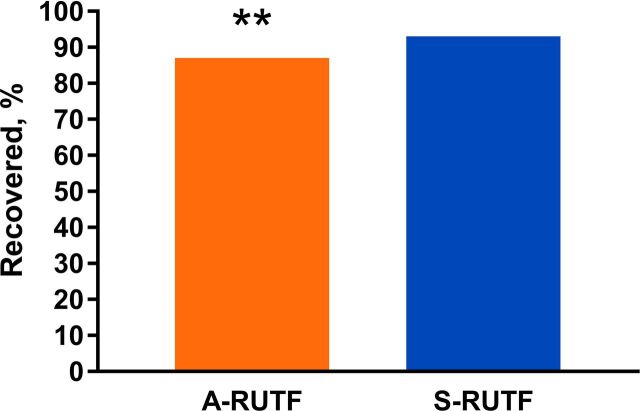
Comparison of Recovery Rates Between A-RUTF and S-RUTF Among Ghanaian Children With Moderate Acute Malnutrition Abbreviations: A-RUTF, alternative ready-to-use therapeutic food; S-RUTF, standard ready-to-use therapeutic food. Intention-to-treat analysis used (Fisher's exact test, **P<.01).

Children with MAM who received A-RUTF were less likely to recover, according to an ITT analysis (Figure 3). For children with MAM, rates of weight and MUAC gain were similar between groups (Table 4). Mortality rates were low, with only 5 (0.4%) MAM or SAM children dying.

A total of 184 (14.5%) of children did not complete the study and were classified as defaults (Table 4). The relative risk for defaulting if the child was enrolled with SAM compared with those with MAM was 3.39 (CI 95%=2.53 to 4.53). Of the children who defaulted, 116 (63.0%) received A-RUTF compared with 68 (37.0%, *P*<.001) who received S-RUTF. Considering the SAM children who defaulted, 68 of 101 (67.3%) did so before the 4-week follow-up, and 7 of 101 (6.9%) did so after the 8-week follow-up. Only 26 of 101 (25.7%) attained an MUAC >11.4 cm, indicative of improvement from SAM to MAM.

Coverage surveys were conducted throughout catchment areas in February 2018. During the survey, the data collection teams assessed a total of 560 children. Among these children, 11 (2.0%) had SAM and 52 (9.3%) had MAM. The coverage of SAM children was 7 of 11 (63.6%) and MAM children was 18 of 52 (34.6%). Mothers were asked if they were aware there was a treatment program in their community, and 8 of 11 (72.7%) of mothers with SAM children and 28 of 52 (53.8%) of mothers with MAM children responded positively.

The cost of A-RUTF used per MAM child recovered was US$7.07, while for S-RUTF the cost was $8.20 (16% higher). The cost of A-RUTF per SAM child recovered was $28.72, while for S-RUTF this was $28.48, a similar amount.

The cost of A-RUTF used per MAM child recovered was US$7.07 and $8.20 for S-RUTF (16% higher).

Untargeted metabolomics of A-RUTF and S-RUTF showed that among the 26 unique metabolites found in A-RUTF, 5 were isoflavones, consistent with the addition of soy products in A-RUTF; while S-RUTF had only 9 unique metabolites, and most were likely to be minor components of the food emulsifier or peanuts (Table 5). No xenobiotics were found in the A-RUTF that were also not present in the S-RUTF.

**Table 5.** Untargeted Metabolomic Assessment of A-RUTF and S-RUTF

Metabolite Class	Specific Metabolites Identified Only in Alternative RUTF	Pathobiological Significance
Phosphatidylcholines	1,2-Dipalmitoleoyl-sn-glycero-3-phosphocholine 1-*O*-Hexadecyl-2-deoxy-2-thio-*S*-acetyl-sn-glyceryl-3 phosphorylcholine 1-Stearoyl-2-myristoyl-sn-glycero-3-phosphocholine 1-Palmitoyl-2-docosahexaenoyl-sn-glycero-3-phosphocholine PC(O-16:0/16:1) PC(18:1/20:2) PC(18:0/20:4) Palmitoyleicosapentaenoyl phosphatidylcholine	Major component of most biological membranes, found in soy foods
Phosphoethanolamines	2-Arachidonoyl-1-palmitoyl-sn-glycero-3-phosphoethanolamine 2-Linoleoyl-1-palmitoyl-sn-glycero-3-phosphoethanolamine	Ethanolamine derivative of phospholipids
Cholesterols	4-Cholestenone 7-Oxocholesterol 4-Beta-hydroxycholesterol 4-acetate	Oxidized forms of cholesterol, the likely source in RUTF is dairy products
Ceramides	Ceramide (18:1/16:0) *N*-Palmitoyl-d-sphingosine	A lipid component of cell membranes that enhances membrane rigidity and facilitates cell signaling through the membrane
Phytosterols	Cholestan-3-one Dihydrodaidzein	Plant-derived sterols typically found in soy products
Isoflavones	Genistin Glycitin 6″-*O*-Acetylgenistin 6″-*O*-Acetylglycitin Daidzin	Isoflavonoid compounds almost entirely derived from legume species, interact with estrogen receptors
Vitamins	Flavine mononucleotide	Form of riboflavin
Glucosyl glucose	(3beta,5xi,9xi,18xi,22beta)-22,25-Dihydroxyolean-12-en-3-yl 6-deoxy-alpha-l-mannopyranosyl-(1->2)-beta-d-xylopyranosyl-(1->2)-beta-d-glucopyranosiduronic acid	A small carbohydrate component of cellulose
**Specific metabolites identified only in standard RUTF**		
Phosphatidylcholines	1-Palmitoyl-2-stearoyl-sn-glycero-3-phosphocholine 1-Docosahexaenoyl-2-stearoyl-sn-glycero-3-phosphocholine 1-Palmitoyl-2-oleoyl-sn-glycerol	Major component of most biological membranes
Lipids	Erucic acid Glycerol 1-myristate	Minor components of edible oils; fatty acid and a monoglyceride
Phenylpropranoids	14-(Methylpentadecanoylamino)-3-phenylpropanoic acid 3,5-Dimethoxy-4-hydroxycinnamic acid 3-Hydroxy-4-methoxycinnamic acid	Food additive made from cinnamic acid and a natural product in coffee and tea
Phenylethylamide	Phenylethylamide 359	Flavoring agent, naturally occurs in peanut

Abbreviations: A-RUTF, alternative ready-to-use therapeutic food; S-RUTF, standard ready-to-use therapeutic food.

## DISCUSSION

An acute malnutrition treatment program was successfully instituted at 29 rural sites in Brong Ahafo, where the prevalence of acute malnutrition was high and a coverage estimate met those typically reported as well as international standards.^23^^,^^24^ Unexpectedly, A-RUTF was not equivalent to S-RUTF in the treatment of SAM or MAM in Ghana in this randomized, double-blind, clinical, controlled trial compared with an ITT analysis. The primary compositional differences were that sorghum and soy were used in A-RUTF in place of some of the peanut paste in S-RUTF and a large portion of the dried skim milk in S-RUTF was replaced with whey protein in A-RUTF.

Unexpectedly, A-RUTF was not equivalent to S-RUTF in the treatment of SAM or MAM in this trial.

The trial was limited by the large number of children who were lost to follow-up. Their outcomes were unknown; however, lost to follow-up or “default” was regarded as a negative outcome, following other RUTF trials and international standards.^4^^,^^21^ Concerted efforts to seek malnourished children in Malawi who were lost to follow-up found that death or hospitalization occurred at about twice the rate as in those who reached a definitive outcome.^4^ There were no differences between the characteristics of children lost to follow-up compared with those who reached a definitive outcome in this study (data not shown).

We used ITT analyses, which are considered the strongest approach for randomized clinical trials to ensure unbiased comparisons among the treatment groups. If a per protocol analysis had been conducted on these SAM data from Ghana, recovery rates would have been 92% and 96% for A-RUTF and S-RUTF, respectively (*P*>.05). If we assume that half of the children lost to follow-up had a definitive negative outcome, then recovery rates for SAM would have been 79% and 87% for A-RUTF and S-RUTF, respectively (*P*=.05). This study is one of very few published clinical trials treating acute malnutrition in Ghana. Previously, a treatment trial of SAM in the Upper East region found a recovery rate of 71% (95% CI=68.0% to 76.0%) and default at 28% (95% CI=24.0% to 32.0%),^25^ results that are similar to our findings from Brong Ahafo. While we believe that our data support the conclusion that A-RUTF is inferior to S-RUTF, this conclusion is tempered by uncertainty from children defaulting.

Our data support that A-RUTF is inferior to S-RUTF, but the conclusion may be affected by defaulting.

Our findings showed that the children receiving A-RUTF enrolled on both SAM and MAM criteria were more likely to default, and we do not have the information to explain why that occurred. In response to a question asked of every caregiver on every return visit, only 3 caregivers overall remarked that their children did not like consuming the RUTF. Most defaulting occurred after 1 or 2 visits. Because of the randomized trial design, we conclude that defaulting is caused by A-RUTF, rather than coincidental circumstances.

Formal acceptability testing was conducted in a crossover design using the RUTFs daily for a week in MAM children. A-RUTF and S-RUTF showed similar amounts consumed, respectively (93% and 92%, *P*>.05) and similar liking scores attributed by the mother (3.5 and 4.1, *P*>.05), and there were no differences in adverse effects.^12^ It is possible children did not like the A-RUTF equivalently over a longer period of time, which led caregivers to not return; however, most defaulting was seen during the first few weeks of treatment. It is unlikely that the whey substituted for milk resulted in the inferior outcomes among the A-RUTF group because RUTF formulations have previously used whey with noninferior outcomes. The acceptability study results, timing of defaulting, and surveys of returning caregivers all indicate that organoleptic inferiority of A-RUTF did not result in the reduced recovery rate.

A study in Malawi found that a milk-free soya, maize, sorghum, amino acid-supplemented RUTF was not inferior to S-RUTF in treating children with SAM.^26^ This study was conducted in a more controlled environment with study participants returning daily to “daycare sites” for supervised feeding. A Zambian study used a similar sorghum RUTF without amino acids in a noninferiority trial and concluded that sorghum RUTF was inferior to S-RUTF in children.^27^ A possible explanation for the inferior outcomes among children receiving the dairy-free sorghum RUTF may have been the acknowledged inferior protein quality compared to S-RUTF. We found that a novel A-RUTF, which also included soy and sorghum, as well as having a similar protein quality as S-RUTF, caused less Ghanaian children to recover from SAM.

Under stressful physiological states, such as during rapid growth, nucleotides are required in the diet for optimal host response.^28^ There are limited data describing the nucleotide content in foods; purine tables are most frequently used to estimate nucleotide content. Grains, such as sorghum, have a low purine content,^29^^,^^30^ which suggests that A-RUTF had a lower nucleotide content than S-RUTF. However, we were not able to detect differences in nucleotide content between the foods in the untargeted metabolomics assays.

Our cost data indicate for SAM that A-RUTF and S-RUTF are similar per child recovered, in spite of a 14% cost reduction per kilogram for the A-RUTF. No savings would be realized by using A-RUTF compared to S-RUTF in SAM.

While A-RUTF and S-RUTF met international specifications for nutrient content, the greater amounts of protein and fat in S-RUTF compared to A-RUTF led to greater rates of weight gain, but these seem unlikely to be important factors in increasing defaulting. RUTF specifications were determined on the basis of expert opinion, not clinical evidence; thus, protein and fat requirements may not be optimal. Some form of food intolerance may possibly have occurred with A-RUTF, resulting in greater default rates. This problem has been observed by the senior author in the past with RUTF made with chickpea in Africa.

With regard to the bioactive metabolites in A-RUTF compared to S-RUTF, the presence of isoflavonoids might have contributed to the poorer outcome. Isoflavonoids have metabolic effects to reduce lipogenesis, which is often thought to be an advantage for healthy consumers in the developed world; however, in this population of acutely malnourished children, this would not be the case.^31^^,^^32^ No xenobiotics or toxins were found in A-RUTF.

The sum of the evidence presented here indicates that A-RUTF is inferior to S-RUTF; it causes lower recovery in SAM and MAM, as well as lower rates of weight and MUAC gain in SAM. It is most important that RUTF facilitate recovery in SAM because SAM causes the most deaths. The certainty of this evidence is tempered by the observation that most failures in our trial were the result of defaulting, and the definitive outcomes in those cases are unknown. In conclusion, we cannot endorse this A-RUTF as noninferior to S-RUTF, and we recommend caution and further testing before any alternative RUTF is used in an operational setting. These data emphasize the utility of randomized trials to assess different RUTFs that meet international standards to determine equivalence.

## Notes

**Peer Reviewed**

**First Published Online:** June 12, 2019
